# Identification and Validation of a Novel Prognostic Signature Based on Ferroptosis-Related Genes in Ovarian Cancer

**DOI:** 10.3390/vaccines11020205

**Published:** 2023-01-17

**Authors:** Zhe Cheng, Yongheng Chen, Huichao Huang

**Affiliations:** 1 Department of Oncology, NHC Key Laboratory of Cancer Proteomics, Laboratory of Structural Biology, National Clinical Research Center for Geriatric Disorders, Xiangya Hospital, Central South University, Changsha 410008, China; 2 Department of Infectious Disease, NHC Key Laboratory of Cancer Proteomics, Laboratory of Structural Biology, National Clinical Research Center for Geriatric Disorders, Xiangya Hospital, Central South University, Changsha 410008, China

**Keywords:** ferroptosis, prognostic signature, ferroptosis-related genes, ALOX12, ovarian cancer

## Abstract

Background: Ovarian cancer is the most lethal gynecological tumor, with a poor prognosis due to the lack of early symptoms, resistance to chemotherapy, and recurrence. Ferroptosis belongs to the regulated cell death family, and is characterized by iron-dependent processes. Here, comprehensive bioinformatics analysis was applied to explore a valuable prognostic model based on ferroptosis-related genes, which was further validated in clinical OC samples. Methods: mRNA data of normal and ovarian tumor samples were obtained separately from the GTEx and TCGA databases. The least absolute shrinkage and selection operator (LASSO) cox regression was applied to construct the prognostic model based on ferroptosis-associated genes. Expression of ALOX12 in OC cell lines, as well as cell functions, including proliferation and migration, were examined. Finally, the prognostic efficiency of the model was assessed in the clinical tissues of OC patients. Results: A gene signature consisting of ALOX12, RB1, DNAJB6, STEAP3, and SELENOS was constructed. The signature divided TCGA, ICGC, and GEO cohorts into high-risk and low-risk groups separately. Receiver operating characteristic (ROC) curves and independent prognostic factor analysis were carried out, and the prognostic efficacy was validated. The expression levels of ALOX12 in cell lines were examined. Inhibition of ALOX12 attenuated cell proliferation and migration in HEY cells. Moreover, the prognostic value of ALOX12 expression was examined in clinical samples of OC patients. Conclusion: This work constructed a novel ferroptosis-associated gene model. Furthermore, the clinical predictive role of ALOX12 was identified in OC patients, suggesting that ALOX12 might act as a potential prognostic tool and therapeutic target for OC patients.

## 1. Introduction

Ovarian cancer (OC) is the most malignant gynecological tumor, and has a poor prognosis. Due to the lack of characteristic symptoms and biomarkers, patients are often diagnosed in the advanced stages [1]. Standard treatments of OC include cytoreductive surgery, chemotherapy, and immunotherapy [2,3]. Even through great improvement has been achieved in terms of diagnosis and treatments, the survival rates of OC patients remain low, and the five-year survival rate is only 47% [4,5]. By contrast, breast cancer has a five-year survival rate of 85%. OC poses a serious threat to the health of women. Thus, it is imperative to develop efficient biomarkers and prognostic signatures to improve the outcomes for OC patients.

Ferroptosis is an iron-dependent regulated cell death form which is characterized by lethal, iron-dependent lipid peroxidation and modulated by glutathione peroxidase 4 (GPX4) [6,7]. To this end, it differs from apoptosis, autophagy, and necrosis [8,9]. Recently emerging studies have demonstrated that ferroptosis is involved in various pathological states such as tumors, ischemia-reperfusion injury, and nervous system diseases [10,11]. For instance, the protein solute carrier family 7, membrane 11 (SLC7A11) inhibited ferroptosis induced by reactive oxygen species (ROS) and abrogated tumor growth suppression mediated by p53, thereby contributing to tumor progression [12]. Similarly, another group reported that tumor suppressor BRCA1-associated protein 1 (BAP1) protein repressed the expression of SLC7A11 in a deubiquitinating-dependent manner, resulting in accumulation of lipid peroxidation and ferroptosis. This result suggested that BAP1 inhibited tumor progression partly via modulating SLC7A11 and ferroptosis [13]. Alvarez et al. found that cysteine desulfurase (NFS1) protected cells from ferroptosis and promoted tumor growth in lung cancer [14]. All of this research indicates that ferroptosis is associated with tumor progression.

In addition, ferroptosis-related genes have been identified which might serve as potential prognostic signatures in some tumors, including breast cancer, hepatocellular carcinoma (HCC), lung cancer, melanoma, and ovarian cancer [15,16,17,18,19,20,21]. There are several bioinformatic methods to establish prognostic models: linear regression, logistic regression, cox regression, and machine learning. The least absolute shrinkage and selection operator (LASSO) is a regression analysis method with both variable selection and regularization functions. It enhances the prediction accuracy and interpretability of the model. Common and popular methods used in establishing prognostic models are LASSO-COX and LASSO-logistic regression [22,23]. Sun and colleagues constructed and validated a prognostic ferroptosis-related risk score (FRRS) in lung adenocarcinomas, which had a robust performance in predicting tumor outcomes [24]. In breast cancer, researchers utilizing public datasets combined with comprehensive bioinformatic analysis established an efficient prognostic signature containing 11 ferroptosis-associated genes. Furthermore, various immune-related biological processes were gathered to participate in the model [15,25]. Some researchers also reported prognostic prediction signatures constructed with ferroptosis-related genes (FRGs) in OC [26]. However, more importantly, these studies merely included limited FRGs to construct prognostic models, which lacked direct validation in clinical tissues of OC patients.

In this work, we enrolled more FRGs to separate OC samples according to the mRNA expression levels from public resources. Then, a five-gene signature was constructed and validated to have outstanding prognostic efficacy in TCGA, ICGC, and GEO cohorts. Functional analyses showed that immune-related processes might be the underlying mechanisms. Furthermore, we assessed the expression and predictive efficacy of lipoxygenase 12 (ALOX12) in clinical OC tissues. Collectively, this study not only identifies a ferroptosis-related prognostic model, but also provides a potential therapeutic target for OC patients.

## 2. Material and Methods

### 2.1. Data Collection

The mRNA expression profiles and clinical information of 374 OC patients were obtained from the Cancer Genome Atlas (TCGA) database, https://portal.gdc.cancer.gov/ (accessed on 17 December 2021). A total of 88 normal ovarian tissue mRNA expression samples were downloaded from the Genotype-Tissue Expression (GTEx) database on 17 December 2021. External validation data were obtained from the International Cancer Genome Consortium (ICGC) database, https://dcc.icgc.org/ (accessed on 17 December 2021) and the Gene Expression Omnibus (GEO) database, https://www.ncbi.nlm.nih.gov/geo/ (accessed on 17 December 2021). The ICGC data used in this study were obtained from Australian OC patient cohort OV-AU, and the GEO datasets used were GSE13876 and GSE49997. The gene expression profiles were normalized by the scale method that was provided in the “limma” R package (v3.54.0). These datasets were achieved from common databases, and strict guidelines were obeyed according to these databases.

A total of 382 Ferroptosis-related genes were downloaded from FerrDb, http://www.zhounan.org/ferrdb/current/ (accessed on 17 December 2021) and divided into three groups: drivers, suppressors, and markers [27]. The counts of these groups are presented in Table 1. Supplementary Table S1 lists all 382 genes.

### 2.2. Analysis of Differentially Expressed and Prognostic Ferroptosis-Related Genes

We conducted analyses to identify expression of ferroptosis-related differentially expressed genes (DEG) and prognosis-related ferroptosis-related genes separately. To analyze ferroptosis-related gene expression conditions both in normal ovary samples and in OC samples, we first extracted transcriptome data of 374 OC samples from the TCGA database and 88 normal ovary samples from the GTEx database, and then combined the data. The “NormalizeBetweenArrays” instruction of R package “limma” was used to remove batch-specific effects in the process. The DEGs were identified with the criteria of false discovery rate (FDR) < 0.05, |log2 FC|≥ 1 from 382 ferroptosis-related genes. We then prepared the clinical data from the TCGA database and used the “bioconductor” R package (v3.16) to combine gene expression data and survival information. A univariate Cox analysis of each ferroptosis-related DEG’s overall survival was performed, and a *p* value less than 0.05 was defined as the threshold. The “venn” R package (v1.11) was used to draw intersectional genes between ferroptosis-related DEGs and prognostic ferroptosis-related genes. Then, we used the “pheatmap” R package (v1.0.12) to obtain a heatmap that reflected the expression of intersectional genes between normal and tumor tissues. The *p* values and hazard ratios (HR) of intersectional genes were shown in the forest map utilizing “survival” R package (v3.4.0). The protein–protein interaction (PPI) analysis was performed via the STRING database. The correlation network between intersectional genes was analyzed using the “igraph” R package (v1.3.5).

### 2.3. Construction and Validation of a Prognostic Ferroptosis-Related Gene Signature

We screened the heatmap and forest map for genes with consistent expression and hazard ratios in both normal tissues and tumor tissues. Then, a univariate Cox analysis of each ferroptosis-related DEG’s overall survival was conducted with the “glmnet” (v4.1.6) and “survival” R packages. Data on the tumor tissues from the TCGA cohort were used as training datasets. The least absolute shrinkage and selection operator (LASSO) regression were utilized in search of a suitable set of potential genes for the prognostic signature. An optimal penalty parameter λ was selected during the LASSO regression to determine the coefficient of each gene in the risk score formula. A formula for risk score was finally achieved by using a linear combination in the TCGA training datasets. The model was constructed as follows:

$$\mathrm{RiskScore}=\overset{N}{\underset{i=1}{\sum }}\left(\exp*coef\right)$$

*N* is the number of genes, exp stands for the expression value of gene, and *coef* stands for the coefficient of the genes in the analysis. Once the risk score model was achieved, OC patients from the TCGA database were divided into a high-risk group and a low-risk group based on the median risk score. Kaplan–Meier survival curve analysis was conducted to compare the overall survival (OS) time between the two subgroups, and a receiver operating characteristic (ROC) curve was drawn by the “timeROC” R package (v0.4) to evaluate the sensitivity and specificity of the gene signature. Two OC cohorts were utilized to validate the prognostic model: cohort OV-AU from the ICGC database and cohort containing GSE49997 and GSE13876 datasets from GEO database. We calculated the risk score of each patient with the same model and divided the patients into a high-risk group and a low-risk group. OS times were compared between the two subgroups.

### 2.4. Functional Annotation Analysis

Patients from three databases were divided into two groups by their risk score, and Wilcoxon tests were conducted by the “limma” R package to examine differentially expressed genes in the two groups. The screen criteria were FDR < 0.05 and |log2 FC|≥ 1. The “colorspace” (v2.0.3), “stringi” (v1.7.8), and “ggplot2” (v3.4.0) R packages were applied to perform the Gene Ontology (GO) analysis and the Kyoto Encyclopedia of Genes and Genomes (KEGG) pathway enrichment analysis. To quantify the activity and enrichment levels of immune cells, functions, or involved immune pathways in the cancer samples, we conducted single-sample Gene Set Enrichment Analysis (ssGSEA) using the “BiocManager” (v1.30.19), “limma”, “GSVA” (v1.46.0), and “GSEABase” (v1.60.0) R packages. We first conducted standardization in order of the gene expression quantity, then the enrichment scores (ES) were calculated by empirical cumulative distribution function. These would later be transformed to achieve abundant immune cell infiltration. Based on the results, correlation analysis of immune scoring was conducted to determine the differences in immune roles between the high-risk and low-risk groups.

### 2.5. Cell Culture

Cells (IOSE80, A2780 and HEY) were all purchased from American Type Culture Collection (ATCC), (Manassas, USA). IOSE80 and A2780 cells were cultured in RPMI-1640 medium containing 10% fetal bovine serum (FBS) and 1% penicillin/streptomycin. HEY cells were cultured in DMEM medium containing 10% fetal bovine serum (FBS) and 1% penicillin/streptomycin. The culture conditions were 37 °C, 5% CO_2_, and 95% humidity.

### 2.6. Western Blotting

Western blotting was performed as previously described [28]. Briefly, OC cells were lysed as indicated in 0.3% Nonidet P40 (Sigma-Aldrich, 74388, St. Louis, MO, USA) buffer containing 150 mM NaCl and 50 mM Tris-HCl, pH = 7.5, and complete protease inhibitor cocktail (Roche, 04693132001, Basel, Switzerland). The following primary antibodies were commercially obtained: ALOX12 (Abcam, 211506; 1:1000 working dilution, Eugene, OR, USA) and goat anti-rabbit IgG (H + L) secondary antibody (Immunoway, RS0002; 1:5000 working dilution, Plano, TX, USA).

### 2.7. 5-Ethynyl-2′-Deoxyuridine (Edu) Staining

The proliferation of ovarian cells HEY was investigated with BeyoClick™ EdU-594 Cell Proliferation Detection Kit (Beyotime, C0078S, Shanghai, China) according to the manufacturer’s protocol. Cells were seeded into 6-well plates overnight at 37 °C. After treatment with or without 20 uM ML355 for 8 h, HEY cells were incubated with EdU working solution (10 uM) for 2 h. Then, cells were washed with PBS twice and fixed with 4% paraformaldehyde for 15 min at room temperature. Next, the cells were washed with PBS (Servicebio, G0002, Wuhan, China) containing 3% BSA (Aladdin, 9048-46-8, Shanghai, China). The cells were incubated with PBS containing 0.3% Triton X-100 (Sigma Aldrich, 9036-19-5, Shanghai, China) for 12 min at room temperature, and were then washed twice with PBS containing 3% BSA. Finally, cells were incubated with Click Additive Solution for 30 min in the dark at room temperature, and subsequently stained with 1× Hoechst for nucleus staining. Images were captured with a fluorescence microscope (Leica, Weztlar, Germany). Cells at the DNA replication phase emitted red fluorescence, while the cell nuclei emitted blue fluorescence.

### 2.8. Transwell Assay

HEY cells were treated with or without 20 uM ML355 for 8 h in advance. HEY cells were seeded with 200 μL serum-free DMEM medium at a density of 1 × 10^4^ in the upper chamber, and the lower chamber was filled with 600 μL full medium. After incubation for 24 h, the upper chamber was fixed and stained with crystal violet for 15 min. Then, the cells were fixed with 4% paraformaldehyde for 30 min, and 1% crystal violet was used to stain cells for 30 min. The remaining cells in the upper chamber were slightly wiped off, and images were collected under a positive microscope (Leica, Weztlar, Germany). Finally, the number of cells passing through the chamber was counted in three random fields under the microscope.

### 2.9. Wound Healing Assay

HEY cells were seeded in the 6-well plate at a density of 2.5 × 10^5^ cells per well and treated with or without 20 uM ML355 for 8 h. Then, 10 μL pipetting heads were used to scratch the cells in the plate, and the serum-free medium was replaced to collect images under an inverted microscope (Leica, Weztlar, Germany) at the indicated time. Wound healing rate = (scratch area 0 h-scratch area 12 h)/scratch area 0 h × 100%.

### 2.10. Immunohistochemical (IHC)

A tissue microarray of Chinese ovarian cancer was purchased from Shanghai Outdo Biotech Co., Ltd (Shanghai, China). The IHC staining of ALOX12 (Abcam, Catalog No.1:50 working dilution) was performed following the manufacturer’s protocol. The tissue microarray was boiled in citrate buffer (pH 6.0) for 10 min for antigen retrieval. The staining intensity score was defined by two independent experienced pathologists as follows: negative (0), weak (1), moderate (2), and strong (3). Percentage scores were defined as 1 (1–25%), 2 (26–50%), 3 (51–75%), and 4 (76–100%). We divided the tissues into high (score ≤ 6) and low (score > 6) groups according to the final score. Final score = intensity score multiplied by percentage score. The expression and clinicopathological characteristics of ALOX12 in ovarian cancer were analyzed by IBM SPSS Statistics 26. The correlation between ALOX12 expression and overall survival was analyzed based on the complete clinical information of the OC samples using the “survival” and “survminer” R packages.

### 2.11. Statistical Analysis

Riskplot was conducted using the “pheatmap” R package. Principal Component Analysis (PCA) and t-distributed stochastic neighbor embedding (t-SNE) analyses were conducted using the “Rtsne” (v0.16) and “ggplot2” (v3.4.0) R packages to visualize the statistics. Univariate and multivariate cox regression analyses were conducted by the “survival” R package. All statistical analysis were conducted based on R (v4.0.2). Two groups were compared with Prism software (GraphPad 6.01) using a two-tailed unpaired Student’s *t*-test. Clinical pathological parameters were analyzed with Statistical Product Service Solutions (SPSS 26.0) using a chi-square test. Statistical significance is indicated with asterisks (*). A two-sided *p* value of <0.05 was considered statistically significant (* *p* < 0.05, ** p < 0.01, *** *p* < 0.001).

## 3. Results

### 3.1. Identifying Prognostic Ferroptosis-Related Genes in OC

**Figure 1:**
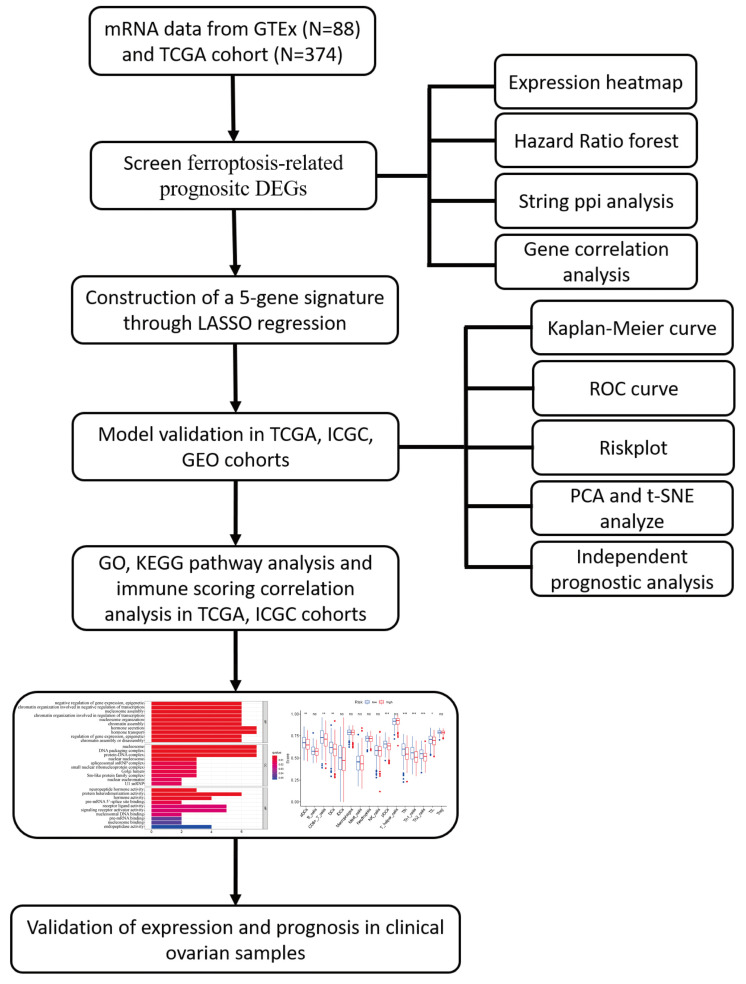
Workflow diagram of data collection and analysis (* *p* < 0.05, ** *p* < 0.01, *** *p* < 0.001).

**Figure 2:**
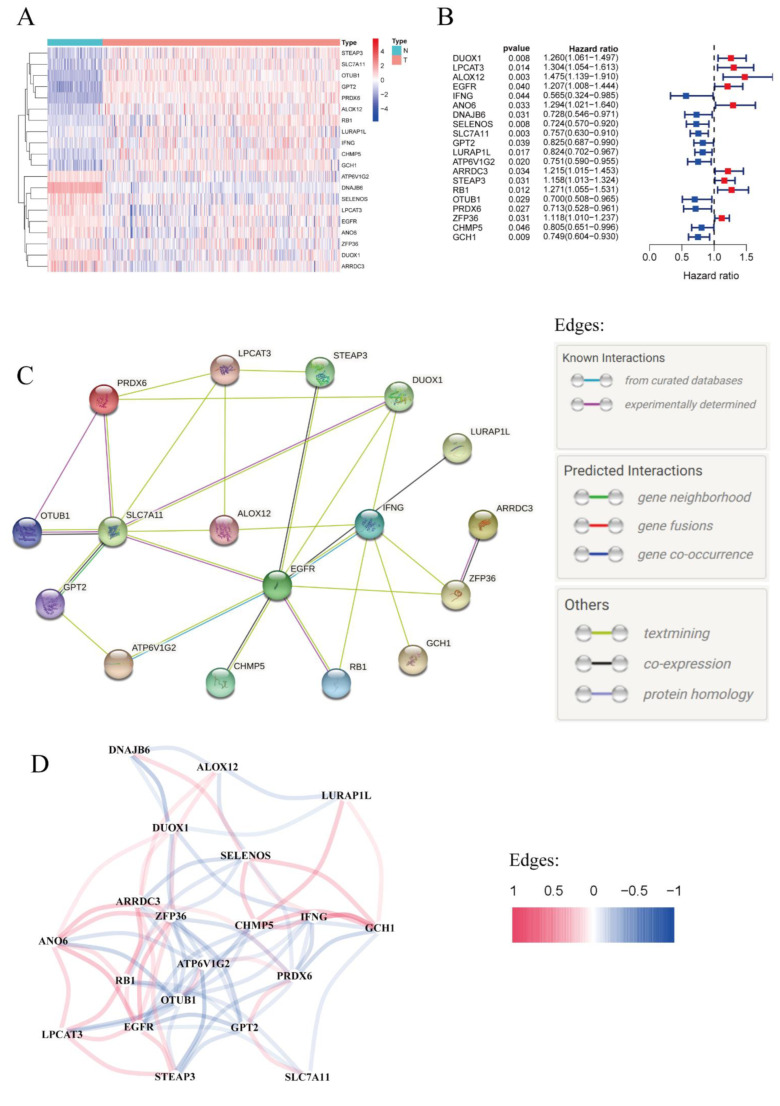
Identification of prognostic ferroptosis-related genes in OC. (**A**) Heatmap reflecting expression levels of prognostic ferroptosis-related genes. Type: N, normal tissues; T, tumor tissues. (**B**) Forest plot of prognostic ferroptosis-related genes. (**C**) PPI network, downloaded from the STRING database, that indicates known interactions and possible interactions among the candidate genes. (**D**) Correlation network between intersectional genes.

The workflow chart of this study is shown in Figure 1. To systematically analyze the differentially expressed ferroptosis-related genes in ovarian cancer tissues and normal tissues, four public databases were utilized to carry out the research. In total, 88 normal ovarian tissues from the GTEx database and 374 OC tissues from the TCGA database were included. The mRNA expression data as well as clinical files were merged and normalized for further comparison using the corresponding R packages. In total, 382 ferroptosis-related genes were analyzed in the present work, and the corresponding mRNA expression data were extracted for further analyses. Eventually, we identified 20 prognostic ferroptosis-related differential expressed genes (DEGs): DUOX1, LPCAT3, ALOX12, EGFR, IFNG, ANO6, DNAJB6, SELENOS, SLC7A11, GPT2, LURAP1L, ATP6V1G2, ARRDC3, STEAP3, RB1, OTUB1, PRDX6, ZFP36, CHMP5, and GCH1. The expression levels of these 20 ferroptosis-related DEGs were visualized via a heatmap (Figure 2A). As shown in Figure 2B, the forest plot depicted univariate Cox regression analysis of the 20 genes. According to the results, these genes were divided into two subgroups: 11 protective genes with hazard ratios (HR) less than 1 (IFNG, DNAJB6, SELENOS, SLC7A11, GPT2, LURAP1L, ATP6V1G2, OTUB1, PRDX6, CHMP5, and GCH1), and 9 risk genes with HR more than 1 (DUOX1, LPCAT3, ALOX12, EGFR, ANO6, ARRDC3, STEAP3, RB1, and ZFP36). Next, we performed PPI analysis using the STRING database. The overall network of PPI is displayed in Figure 2C, indicating that EGFR, SLC7A11, and IFNG are hub genes. The possible association of these 20 genes was also analyzed (Figure 2D). Figure 2D showed that ALOX12 had negative correlations with DNAJB6 and SELENOS, but had positive correlations with ARRDC3 and ZFP36.

### 3.2. Construct a Prognostic Model in the TCGA Cohort

**Figure 3:**
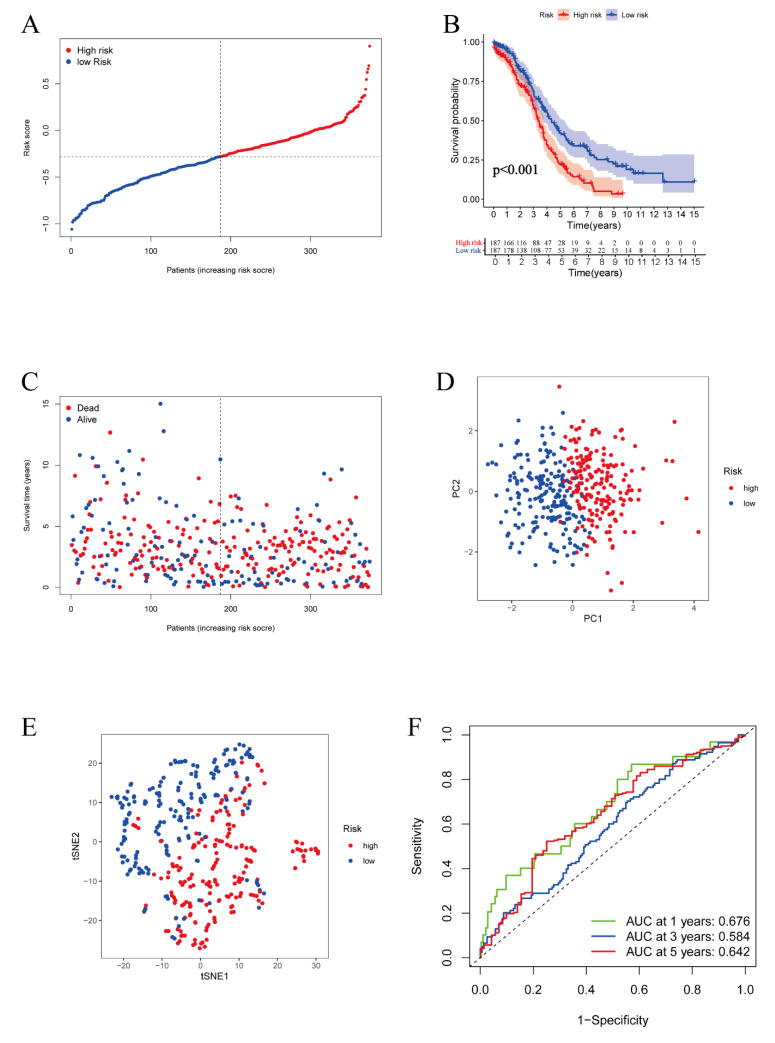
Construction of a prognostic signature in the TCGA cohort. **(A**) Distribution of the risk score in the TCGA cohort. (**B**) Kaplan–Meier survival curve for OS of ovarian cancer in the TCGA cohort. (**C**) Distribution of survival status and risk score of each patient. (**D**) PCA plot of the TCGA cohort by 5 genes. (**E**) t-SNE analysis in the TCGA cohort by 5 genes. (**F**) ROC curve demonstrating the predictive efficiency of the 5-gene prognostic signature in the TCGA cohort.

To establish a prognostic signature according to the expression levels of the 20 genes mentioned above, LASSO cox regression and multivariate cox analyses were performed. Five genes with consistent expression and hazard ratios on the heatmap and the forest map, in both normal tissues and tumor tissues, were screened out. Eventually, a five-gene model was identified based on the optimal value of λ (Supplementary Figure S1). The hazard model was constructed as a formula: Risk score = (0.2686 * expression level of ALOX12) + (0.1413 * expression level of STEAP3) + (0.1787 * expression level of RB1) − (0.2613 * expression level of DNAJB6) − (0.2126 * expression level of SELENOS). Patients from the TCGA cohort were divided into high-risk and low-risk subgroups based on the risk score calculated by the formula (Figure 3A). As expected, the Kaplan–Meier survival curve showed that the high-risk group had a poorer survival rate than the low-risk group (*p* = 1.983 × 10^−05^) (Figure 3B). Consistently, patients with high risk scores had shorter survival times compared to those with low risk scores (Figure 3C). Furthermore, we assessed the predictive efficiency of the five-gene prognostic signature by the PCA and t-SNE analyses as well as the ROC curve. As displayed in Figure 3D,E, PCA and t-SNE analyses demonstrated that our prognostic model could effectively stratify the patients into high or low-risk groups. The ROC curve depicted the predictive overall survival value of our signature in ovarian cancer. Meanwhile, as presented, the area under the curve was 0.676 at 1 year, 0.584 at 3 years, and 0.642 at 5 years (Figure 3F).

### 3.3. Validating the Prognostic Ferroptosis-Related Signature in the ICGC and GEO Cohorts

**Figure 4:**
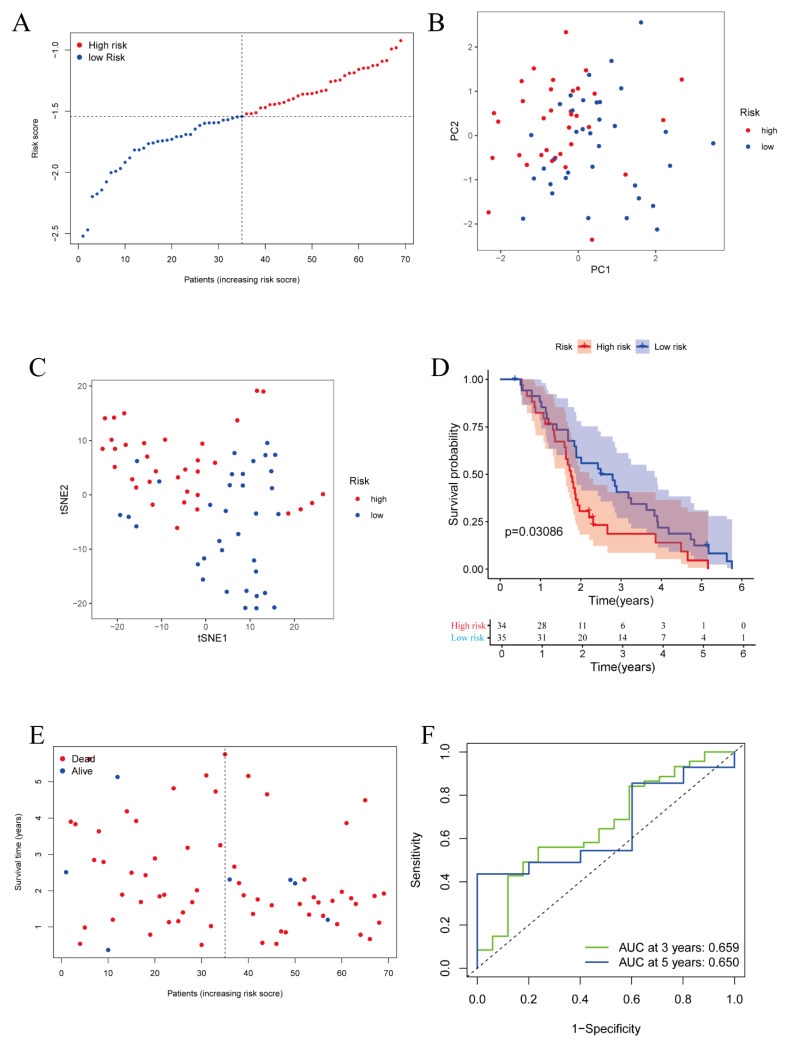
Validation of the prognostic signature in the ICGC cohort. (**A**) Distribution of the risk score in the ICGC cohort. (**B**) PCA plot of the ICGC cohort by 5 genes. (**C**) t-SNE analysis in the ICGC cohort by 5 genes. (**D**) Kaplan–Meier survival curve for OS of ovarian cancer in the ICGC cohort. (**E**) Distribution of survival status and risk score of each patient. (**F**) ROC curve demonstrating the predictive efficiency of the 5-gene prognostic signature in the ICGC cohort.

ICGC and GEO datasets were utilized to validate the prognostic value of the five-gene model constructed in the TCGA database. A total of 69 patients from the ICGC database were separated into a high-risk group and a low-risk group (34 and 35 samples, respectively) based on the risk score formula mentioned previously (Figure 4A). Both PCA and t-SNE analyses indicated that the risk score model effectively separated the patients into two directions (Figure 4B,C). Similarly, the Kaplan–Meier survival curve showed that patients in the high-risk group had worse OS rates than those in the low-risk group (Figure 4D), and the survival status results displayed that the risk score correlated with the survival condition of the patients. (Figure 4E). Moreover, the time-dependent ROC curve demonstrated that the risk score model also showed predictive efficiency as well as accuracy in the ICGC cohort, and the AUC of the ROC curve reached 0.659 at 3 years and 0.650 at 5 years, respectively (Figure 4F). These data showed that the gene model was valid and possessed significant predictive value; in addition, cases could be effectively divided into two subgroups.

**Figure 5:**
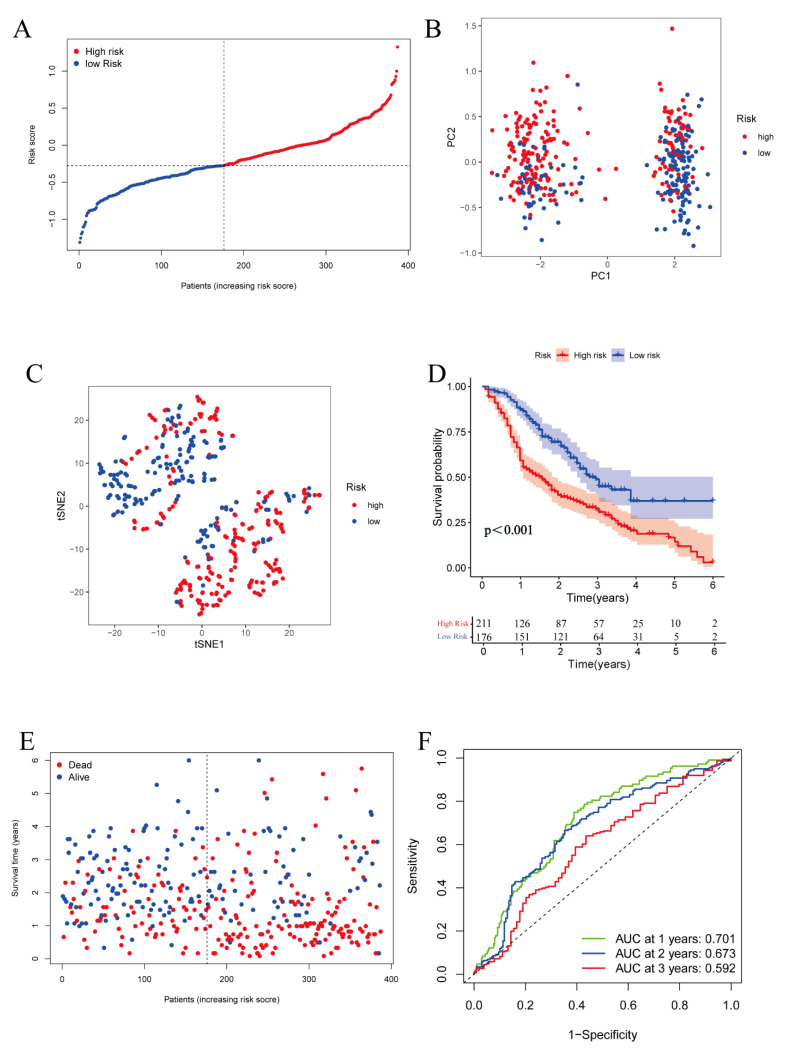
Validation of the prognostic signature in the GEO cohort. (**A**) Distribution of the risk score in the GEO cohort. (**B**) PCA plot of the GEO cohort by 5 genes. (**C**) t-SNE analysis in the GEO cohort by 5 genes. (**D**) Kaplan–Meier survival curve for OS of ovarian cancer in the GEO cohort. (**E**) Distribution of survival status and risk score of each patient. (**F**) ROC curve demonstrating the predictive efficiency of the 5-gene prognostic signature in the GEO cohort.

Furthermore, we assessed the prognostic value of this signature in two GEO datasets. Similarly to the results observed in the TCGA and ICGC databases, the samples were also separated into two groups: 211 high-risk samples and 176 low-risk samples (Figure 5A). The risk score model effectively separated the patients into two groups via PCA and t-SNE analyses (Figure 5B,C). In addition, the data showed that these two groups had a statistical difference in survival probability (Figure 5D). Meanwhile, the survival status plot displayed that the survival conditions of patients were correlated with the risk scores calculated by the risk score model (Figure 5E). The AUC of the ROC curve was 0.701 at 1 year, 0.673 at 2 years, and 0.592 at 3 years (Figure 5F). In conclusion, these results showed that our risk signature possessed consistent and valid prognostic value, in both the ICGC and GEO databases.

### 3.4. Independent Prognostic Value of the Ferroptosis-Related Five-Gene Signature

**Figure 6:**
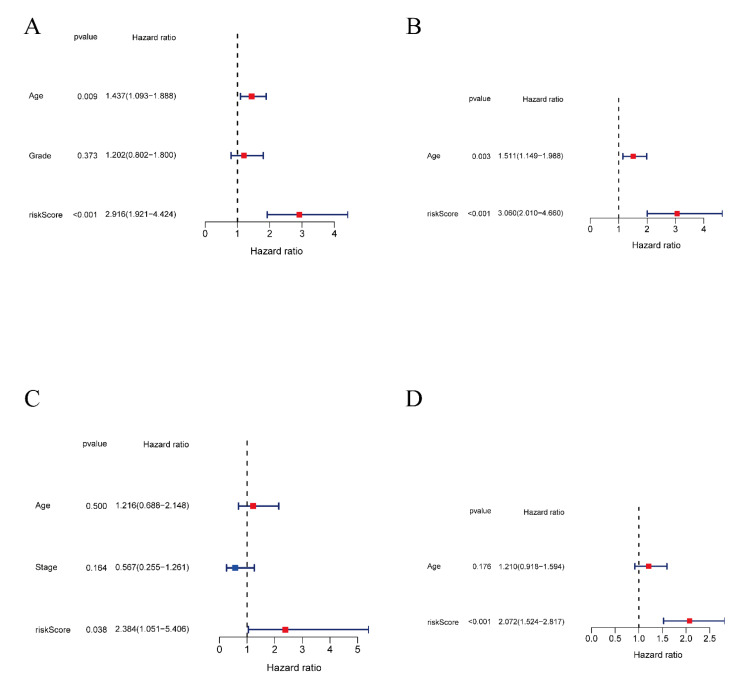
Univariate and multivariate independent prognostic analyses. (**A**) Univariate independent prognostic analysis in the TCGA cohort. (**B**) Multivariate independent prognostic analysis in the TCGA cohort. (**C**) Univariate independent prognostic analysis in the ICGC cohort. (**D**) Univariate independent prognostic analysis in the GEO cohort.

Univariate and multivariate independent prognostic analyses were conducted among the TCGA, ICGC, and GEO databases to examine independent prognostic and clinical values of the model. The univariate cox regression and multivariate cox regression were processed using the “survival” R package. The age threshold was set at 65 years old. The criteria for the control group were an age of no more than 65 years and a pathological grade lower than grade IV. Both univariate and multivariate independent prognostic analyses were carried out in the TCGA database, and the results indicated that risk score was significantly related to overall survival time (HR = 2.916, 95% CI: 1.916 to 4.424; HR = 3.060 95% CI: 2.010 to 4.660, respectively) (Figure 6A,B). Furthermore, in both the ICGC and GEO databases, the univariate independent prognostic analysis also indicated that the risk signature was a reliable independent prognostic factor for OS in ovarian cancer (HR = 2.384, 95% CI: 1.051 to 5.406; HR = 2.072, 95% CI: 1.524 to 2.817, respectively) (Figure 6C,D). Collectively, these data revealed that the five-gene prognostic signature could predict the prognosis of OC patients independently, thereby possessing significant clinical value for OC treatment.

### 3.5. Functional Analyses of the Ferroptosis-Related Five-Gene Signature

**Figure 7:**
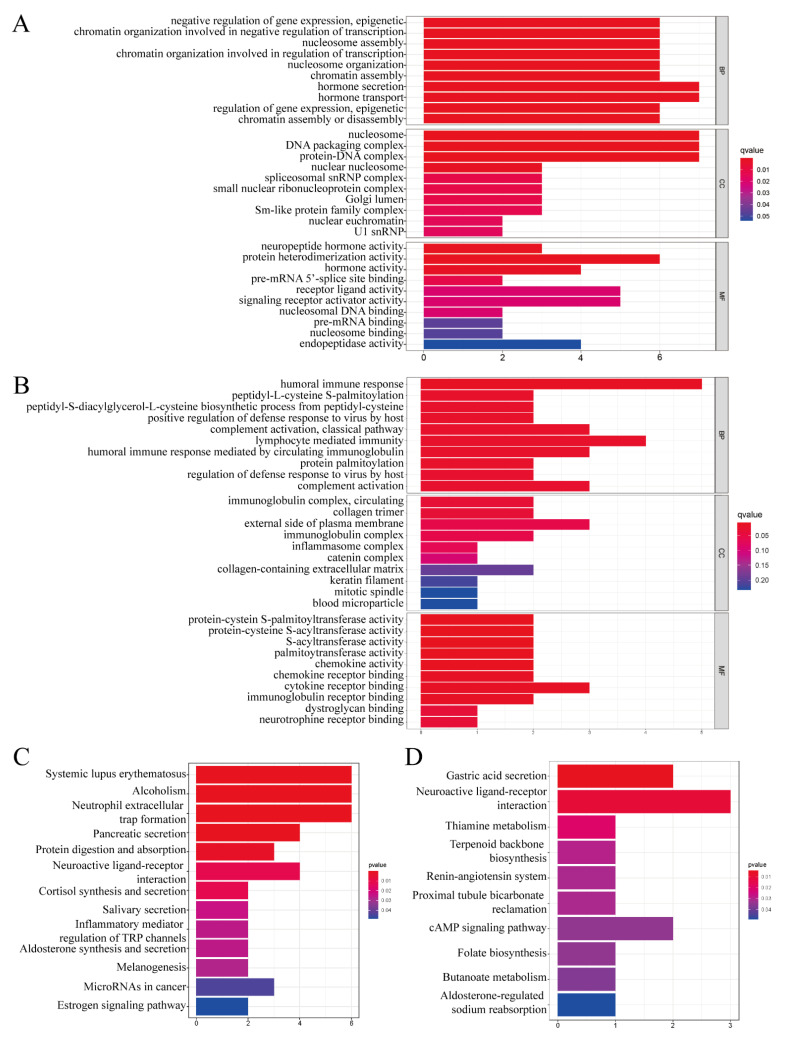
Gene enrichment analysis in the TCGA and ICGC cohorts. (**A**) Go annotation analysis in the TCGA cohort. (**B**) Go annotation analysis in the ICGC cohort. (**C**) KEGG annotation analysis in the TCGA cohort. (**D**) KEGG annotation analysis in the ICGC cohort.

To obtain a global view of biological roles and processes of the risk score, GO and KEGG annotation analyses were carried out using the ferroptosis-related DEGs in both the TCGA and ICGC databases. Molecular function analysis hinted that some immune-related processes were enriched, such as humoral immune response, complement activity, lymphocyte-mediated immunity, and immunoglobulin complex circulation (Figure 7A,B). KEGG pathway analysis also indicated that the DEGs were closely linked to some immune-associated pathways (Figure 7C,D). Molecular function and pathway results synergistically demonstrated that the DEGs mainly participated in immunity disorders, which may be the underlying predictive prognostic mechanism of the risk score model in ovarian cancer patients.

**Figure 8:**
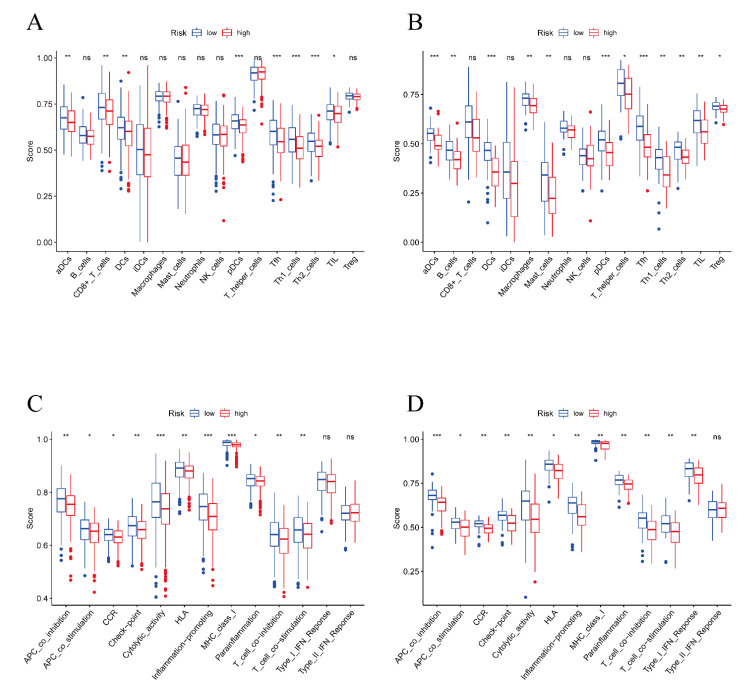
ssGSEA scores between different risk groups in the TCGA cohort and ICGC cohort. (**A**) The abundance of immune cells in the TCGA cohort. (**B**) The abundance of immune cells in the ICGC cohort. (**C**) The immune function analysis in the TCGA cohort. (**D**) The immune function analysis in the ICGC cohort. (* *p* < 0.05; ** *p* < 0.01, *** *p* < 0.001, ns: not significant).

In order to better determine the differences in immune cell characteristics between the high-risk and low-risk groups, we conducted ssGSEA analysis to assess immune cell infiltration and related functions in the TCGA and ICGC databases. The abundance of immune cells was evaluated, and the results demonstrated that the abundance of aDCs, DCs, pDCs, Tfh, Th1, Th2, and TIL cells was dramatically lower in the high-risk group (Figure 8A,B). In addition, the immune cell function analysis also revealed that some vital immune related pathways, including T cell co-stimulation, Type I IFN response, inflammation promoting, and MHC class I processes, were up-regulated in the low-risk group (Figure 8C,D). Taken together, these results suggest that these ferroptosis-related DEGs might exert their prognostic effect by manipulating the activity of immune cells in ovarian cancer.

### 3.6. The Expression and Prognosis Value of ALOX12 Were Evaluated in Clinical Ovarian Samples

**Figure 9:**
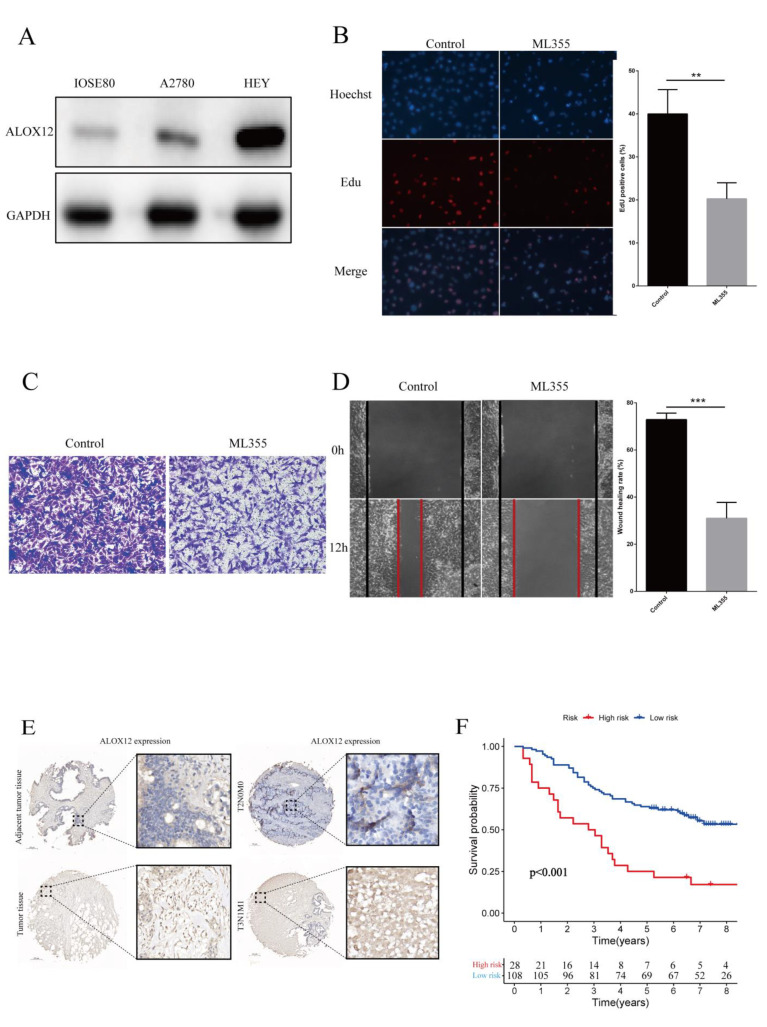
Evaluation of ALOX12 expression and prognosis value in clinical ovarian samples. (**A**) Immunoblot analysis of ALOX12 in OC cells. The whole cell lysates extracted from IOSE80, A2780, and HEY cell lines were subjected to Western blot analysis with the indicated antibodies. (**B**) The proliferative ability of HEY cells was detected by EdU assay. HEY cells were incubated with or without 20 uM ML355 for 8 h (mean ± SD; *n* = 3 biological replicates per group; ** *p* < 0.01; unpaired Student’s *t*-test). (**C**) The migration ability of HEY cells was detected by Transwell assay. HEY cells were incubated with or without 20 uM ML355 for 8 h in advance. (**D**) The migration ability of HEY cells was detected by wound healing assay. HEY cells were incubated with or without 20 uM ML355 for 8 h in advance (mean ± SD; *n* = 3 biological replicates per group; *** *p* < 0.001; unpaired Student’s *t*-test). (**E**) Immunohistochemical staining of ALOX12 protein in clinical ovarian samples. Representative pictures of ALOX12 protein in tumor and adjacent tissues and in different clinical-stage tissues. (**F**) Kaplan–Meier survival curve for OS of ovarian cancer based on ALOX12 expression in the IHC cohort.

ALOX12, as a ferroptosis driver gene, has been reported to be expressed aberrantly in several cancers. However, its functions have not been assessed in ovarian cancer. In this study, we found that ALOX12 was differentially expressed between normal ovarian tissues and ovarian cancer tissues. Heatmap and forest plot results showed that ALOX12 was overexpressed in ovarian cancer tissues with a high hazard ratio. In addition, in the risk score formula, ALOX12 had the largest absolute value of its gene coefficient, indicating that the ALOX12 gene expression level most significantly affected the risk score. ALOX12 reached its maximum weight in the five-gene signature, indicating that ALOX12 might be an important biomarker for predicting ovarian cancer prognosis. To further validate the potential clinical values of ALOX12 in OC, we tested ALOX12 protein expression. The Western blotting assay showed that ALOX12 expression levels were strikingly improved in malignant cell lines (A2780 and HEY cell lines) compared with those in normal cells (Figure 9A).

Then, functions of ALOX12 were explored in HEY cells. A specific ALOX12 inhibitor, ML355 (Selleck, S6557, Houston, TX, USA), was used in this study. Cell functions, including proliferation and migration, were tested after 20 uM ML355 treatment for 8 h by EdU assay, Transwell assay, and wound healing assay. The EdU experiment showed that inhibition of ALOX12 activity reduced cell proliferation ability (Figure 9B). Consistently, both the Transwell assay and the wound healing assay indicated that inhibition of ALOX12 activity attenuated cell migration (Figure 9C,D). The results implied that ALOX12 promoted cell proliferation and migration in HEY cells.

Subsequently, utilizing the cohort of 136 OC tissues, we observed that ALOX12 protein expression was increased in malignant tissues compared with that in adjacent tissues. Moreover, our results demonstrated that ALOX12 statuses were positively associated with node metastasis (*p* < 0.001), distant metastasis (*p* < 0.001), and clinical stages (*p* < 0.05), but not with age or tumor size (Figure 9E, Table 2). Based on the complete clinical information of the OC samples, the correlation between ALOX12 expression and overall survival was analyzed. As displayed in Figure 9F, patients with higher ALOX12 expression possessed a robustly poorer prognosis than those with lower ALOX12 expression. These results collectively supported that ALOX12 may function as a potential prognostic marker and an independent prognostic factor in OC patients.

## 4. Discussion

Evasion of regulated cell death is a pivotal hallmark of cancer. Ferroptosis is a novel cell death type with unique characteristics and roles, participating in physical processes as well as many diseases, including cancer. Different from apoptosis, necrosis, and other forms of RCD, ferroptosis is characterized by iron-dependent lipid peroxidation [29]. Recently, researchers have proven that ferroptosis is closely linked to the initiation, development, and treatment of cancer [30,31]. Hence, exploration of the therapeutic strategies of ferroptosis has garnered enormous interest in the cancer research community. These strategies include the development of ferroptosis-inducing agents (FINs), such as erastin and RSL3, which have been applied to treat tumors alone or in combination with other therapies. However, the studies on ferroptosis are still in the infant stages, especially regarding the exploration of the prognostic function of ferroptosis in tumors, including OC.

OC is a highly malignant gynecological cancer. Although huge efforts have been applied into its investigation, efficient diagnostic and prognostic biomarkers are missing, which places a huge burden on the public health community and encourages deeper research for OC patients. In this work, we explored the 382 well-defined ferroptosis genes in malignant and normal ovarian tissues. Applying constructive analyses, a five-gene ferroptosis-related prognostic signature was conducted and assessed in multiple common datasets. Furthermore, we investigated the biological roles and processes of the prognostic signature and found that immune-associated processes were enriched. Importantly, the expression levels of ALOX12, belonging to the five-gene ferroptosis-related prognostic signature, had a positive correlation with lymph node and distant metastasis as well as the overall survival of OC patients.

Our five-gene ferroptosis-related prognostic signature includes ALOX12, STEAP3, RB1, DNAJB6, and SELENOS, of which ALOX12 and DNAJB6 belong to ferroptosis driver genes, RB1 is a ferroptosis suppressor gene, and STEAP3 and SELENOS are marker genes. ALOX12 acts on polyunsaturated fatty acids, mainly arachidonate, and generates bioactive lipid (12S)-hydroperoxy eicosatetraenoic acid [32]. Lipid production induced by ALOX12 plays an important role in ferroptosis, and prevention of dysregulation of ALOX12 and GPL4 rescues animals from toxicant-induced ferroptosis [33]. ALOX12 has also been found to be involved in p53-mediated tumor suppression, which is independent of the ACSL-4 ferroptosis pathway [34]. In terms of the relationship between ALOX12 and the prognosis of cancer, other groups demonstrated that ALOX12 promoted the malignancy and chemoresistance of tumor cells, including breast cancer and colon cancer. Similarly, we found that ALOX12 was overexpressed in ovarian cancer cells, and inhibition of ALOX12 attenuated the proliferation and migration of ovarian cancer cells. We discovered that ALOX12 expression was substantially higher in malignant samples than in adjacent samples, and was linked to lymph node and distant metastasis, but not to patient age or tumor size in OC samples. Meanwhile, the increased ALOX12 protein expression was associated with the poor prognosis of OC patients, indicating that ALOX12 may serve as a potential risk factor in OC.

STEAP3, as a metalloreductase, converts iron from Fe^3+^ to Fe^2+^ and, therefore, participates in iron homeostasis in tissues. It has been reported that STEAP3 overexpression contributes to apoptosis and inhibits G2/M transition in cell cycle progression. However, other research has shown that STEAP3 participates in the progression of some tumors. Wang L. et al. revealed that STEAP3 was overexpressed and promoted cell proliferation via the RAC1-ERK-STAT3 signaling pathway in HCC [35]. The complex and varied functions of STEAP3 hint at its critical roles in tumors. In this research, we found that STEAP3 was overexpressed in OC and verified that it might be a promising prognostic marker for OC patients.

The retinoblastoma susceptibility gene (Rb1), as a tumor repressor gene, has critical effects on multiple cellular processes, such as ferroptosis, apoptosis, and DNA repair. The inactivation of Rb1 dramatically accelerates the induction of ferroptosis and sensitivity to sorafenib in human hepatocellular carcinoma cells [36]. DNAJB6 is an endogenous anti-aggregation molecular chaperone [37]. Until now, it has not successfully characterized the functions of DNAJB6 or SELENOS in the ferroptosis process. We used the remaining three genes, ALOX12, STEAP3, and RB1, to build a model, and the result showed that it was unable to effectively divide the ICGC patients into two sub-groups. The Kaplan–Meier survival curve for OS in the ICGC cohort turned out to be statistically insignificant (*p* = 0.2607) (Supplementary Figure S2). DNAJB6 was found to promote ferroptosis and to serve as a protective factor in esophageal squamous cell carcinoma tissue [38]. SELENOS is a transmembrane protein located in the endoplasmic reticulum (ER). It is involved in misfolded protein degradation and inflammation response [39]. In our research, the expression of DNAJB6 and SELENOS were decreased in OC samples compared with normal ovarian samples. However, to gain a deeper insight into the roles of DNAJB6 and SELENOS in regulating ferroptosis-related processes, much more work will be carried out in the future.

## 5. Conclusions

In summary, this study constructed and evaluated a five-ferroptosis-related-gene model which can potentially predict the prognosis of OC patients in the common databases. Bioinformatic analyses unraveled that DEGs between high-risk and low-risk subgroups were found to be highly associated with tumor immune status. Furthermore, ALOX12 protein expression was assessed in clinical OC tissues, and was found to be positively associated with tumor lymph nodes and distant metastasis. Our results demonstrate a valuable prognostic risk model which might provide a useful resource for clinical decision-making and novel therapeutic targets for OC patients.

## Figures and Tables

**Table 1:** Ferroptosis-related gene sets achieved from FerrDb.

Gene Sets	Counts
Drivers	150
Suppressors	109
Markers	123

**Table 2:** Expression and clinicopathological characteristics of ALOX12 in ovarian cancer.

Variables	*n*	ALOX12 Expression		*p* Value
		Low Expression	High Expression	
Age (years)				
≤ 60	103	82	21	0.856
>60	32	25	7	
T				
T1	7	5	2	
T2	33	31	2	0.059
T3	96	72	24	
N				
N0	104	98	6	0.001
N1	32	8	22	
M				
M0	109	97	12	0.001
M1	27	11	16	
Clinical stage				
I + II	40	36	4	0.049
III + IV	96	72	24	

Age information was missing for one patient. Age, TNM stage, and clinical stage were analyzed for statistical difference between the ALOX12 high expression group and the low expression group. Data were analyzed with SPSS using a chi-square test.

## Data Availability

The original contributions presented in the study are included in the article/Supplementary Materials. Further inquiries can be directed to the corresponding author.

## Supplementary Materials

The following supporting information can be downloaded at: https://www.mdpi.com/article/10.3390/vaccines11020205/s1, Figure S1: Gene coefficient profiles and partial likehood deviance determined by LASSO regression; Figure S2: Result using ALOX12, STEAP3, and RB1 to build a model; Table S1: List of all 382 ferroptosis-related genes.

## Abbreviations

OC: ovarian cancer; GPX4: glutathione peroxidase 4; SLC7A11: solute carrier family 7, membrane 11; ROS: reactive oxygen species; BAP1: BRCA1 associated protein 1; NFS1: cysteine desulfurase; HCC: hepatocellular carcinoma; TCGA: the Cancer Genome Atlas; ICGC: International Cancer Genome Consortium; GEO: Gene Expression Omnibus; ssGSEA: single sample Gene Set Enrichment Analysis; ALOX12: lipoxygenase 12.
