# Supplementary material for: Identification and Validation of a Novel Prognostic Signature Based on Ferroptosis-Related Genes in Ovarian Cancer
Source: Vaccines (Basel). 2023 Jan 17;11(2):205. doi: 10.3390/vaccines11020205 (PMC9962729; doi:10.3390/vaccines11020205)
Supplement: Supplementary file 1 [file vaccines-11-00205-s001.zip › vaccines-2150442-Figure S1.pdf]

A

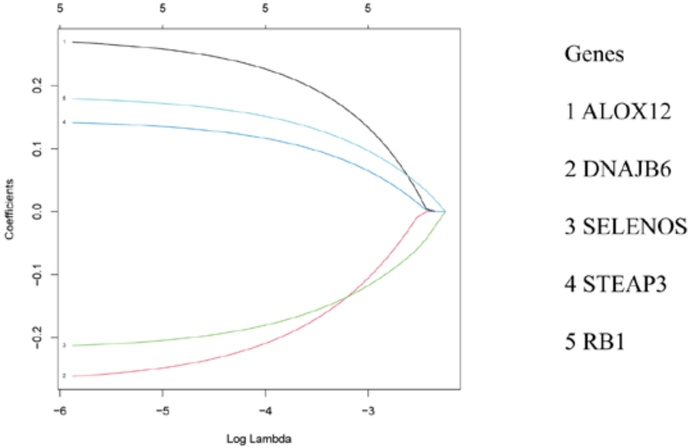

Figure S1A Gene coefficient profiles determined by LASSO regression.

B

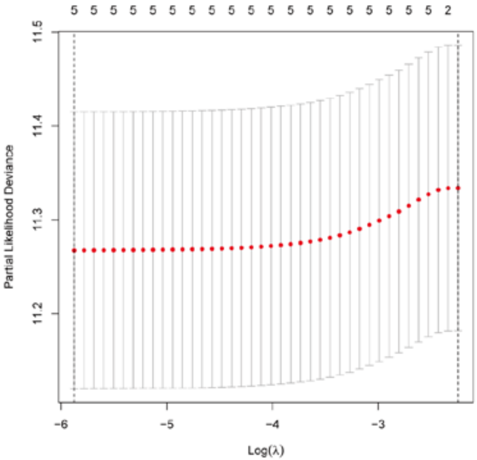

Figure S1B Partial likehood deviance plotted with log (lambda).
