# Supplementary material for: Identification and Validation of a Novel Prognostic Signature Based on Ferroptosis-Related Genes in Ovarian Cancer
Source: Vaccines (Basel). 2023 Jan 17;11(2):205. doi: 10.3390/vaccines11020205 (PMC9962729; doi:10.3390/vaccines11020205)
Supplement: Supplementary file 1 [file vaccines-11-00205-s001.zip › vaccines-2150442-Figure S2.pdf]

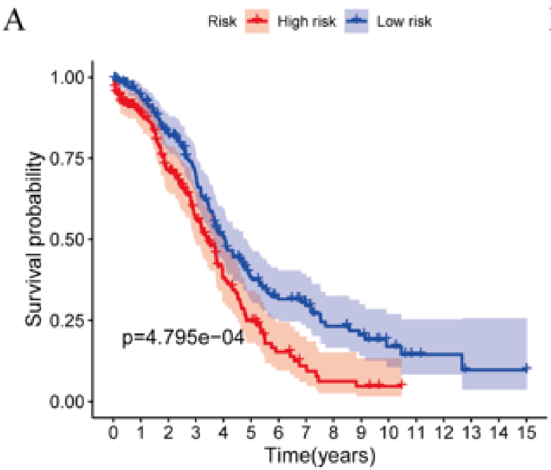

Figure S2A Kaplan-Meier survival curve for OS of Ovarian cancer in the TCGA cohort.

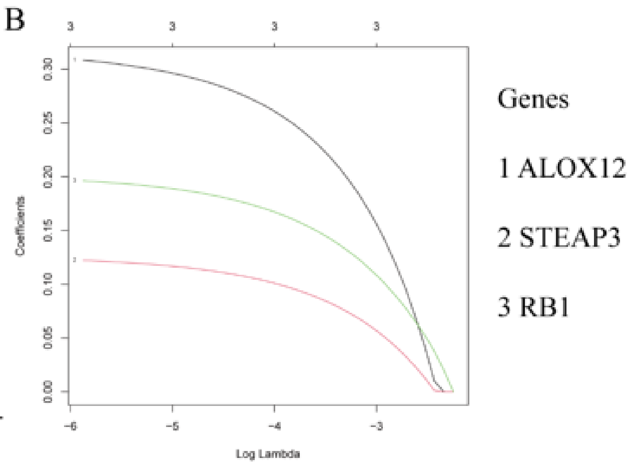

Figure S2B Gene coefficient profiles determined by LASSO regression.

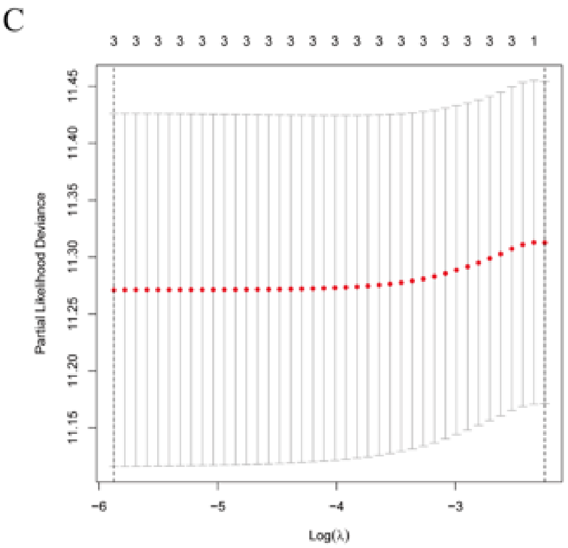

Figure S2C Partial likelihood deviance plotted with log (lambda)

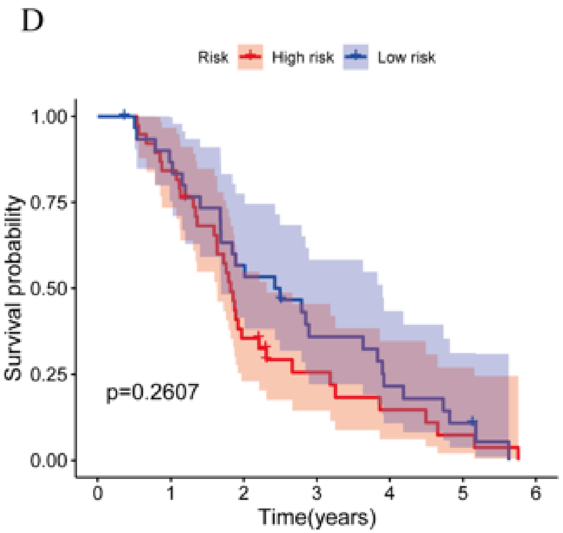

Figure S2D Kaplan-Meier survival curve for OS of Ovarian cancer in the ICGC cohort
